# Developing a two-dimensional model of unprofessional behaviour profiles in medical students

**DOI:** 10.1007/s10459-018-9861-y

**Published:** 2018-11-01

**Authors:** Marianne C. Mak-van der Vossen, Anne de la Croix, Arianne Teherani, Walther N. K. A. van Mook, Gerda Croiset, Rashmi A. Kusurkar

**Affiliations:** 1 Department of Research in Education, VUmc School of Medical Sciences, Amsterdam UMC, PO Box 7057, 1007 MB Amsterdam, The Netherlands; 2 LEARN! Academy, Vrije Universiteit, Amsterdam, The Netherlands; 3 Center for Faculty Educators, School of Medicine, University of California San Francisco, San Francisco, USA; 4 Department of Intensive Care Medicine, Maastricht University Medical Center, Maastricht University, Maastricht, The Netherlands; 5 Faculty of Medical Sciences, University Medical Center Groningen, Groningen, The Netherlands

**Keywords:** Attitude, Consensus, Faculty, Undergraduate medical education, Medical schools, Medical students, Professionalism, Professional misconduct, Unprofessional behaviour

## Abstract

Standardized narratives or *profiles* can facilitate identification of poor professional behaviour of medical students. If unprofessional behaviour is identified, educators can help the student to improve their professional performance. In an earlier study, based on opinions of frontline teachers from one institution, the authors identified three profiles of medical students’ unprofessional behaviour: (1) *Poor reliability,* (2) *Poor reliability and poor insight*, and (3) *Poor reliability, poor insight and poor adaptability*. The distinguishing variable was *Capacity for self*-*reflection and adaptability*. The current study used Nominal Group Technique and thematic analysis to refine these findings by synthesizing experts’ opinions from different medical schools, aiming to develop a model of unprofessional behaviour profiles in medical students. Thirty-one experienced faculty, purposively sampled for knowledge and experience in teaching and evaluation of professionalism, participated in five meetings at five medical schools in the Netherlands. In each group, participants generated ideas, discussed them, and independently ranked these ideas by allocating points to them. Experts suggested ten different ideas, from which the top 3 received 60% of all ranking points: (1) *Reflectiveness* and *adaptability* are two distinct distinguishing variables (25%), (2) The term *reliability* is too narrow to describe unprofessional behaviour (22%), and (3) Profiles are dynamic over time (12%). Incorporating these ideas yielded a model consisting of four profiles of medical students’ unprofessional behaviour (*accidental behaviour*, *struggling behaviour*, *gaming*-*the*-*system behaviour* and *disavowing behaviour*) and two distinguishing variables (*reflectiveness* and *adaptability*). The findings could advance educators’ insight into students’ unprofessional behaviour, and provide information for future research on professionalism remediation.

**Electronic supplementary material:**

The online version of this article (10.1007/s10459-018-9861-y) contains supplementary material, which is available to authorized users.

## Introduction

Evaluating medical students’ professional performance is a difficult and sensitive activity (Veloski et al. 2005; Ginsburg et al. 2009). It often results in educators’ *failure to fail* underperforming students (Yepes-Rios et al. 2016). When underperforming students are not identified, they cannot be offered assistance to help them improve their performance (Ellaway et al. 2017). It would be important for undergraduate medical education to create research-based tools to facilitate identification of poor professional performance of medical students, and help teachers recognize students who may benefit from extra guidance in order to overcome any difficulties (Arnold 2002; Hodges et al. 2011). Outcomes of the current study could guide medical educators in identification of those students who are expected to benefit from professionalism remediation activities, and those who are considered unfit for practice.

Early detection and remediation of poor performance of medical students is essential (Papadakis et al. 2005). Current literature has focused on strategies to detect students who behave unprofessionally, aiming to provide feedback to these learners and to identify students who need remediation. Attention has been given to descriptors and categories of students’ unprofessional behaviours, which include *poor engagement*, *lack of integrity*, *poor interaction with others*, and *poor self*-*awareness,* including *not responding to feedback* (Teherani et al. 2009; Mak-van der Vossen et al. 2017). Other examples of such detection strategies focus on the egregiousness of the behaviours (Cullen et al. 2017), on attributions for behaviours (Ginsburg et al. 2009), on underlying problems (Hays et al. 2011), on predictors of poor academic outcomes (Guerrasio et al. 2014), and on students’ characteristics that form risk factors for professional misconduct (Yates 2014; Yates and James 2010). In these studies, the unprofessional behaviours are mostly approached as isolated events, rather than patterns comprising a combination of behaviours and surrounding incidents.

**Fig. 1:**
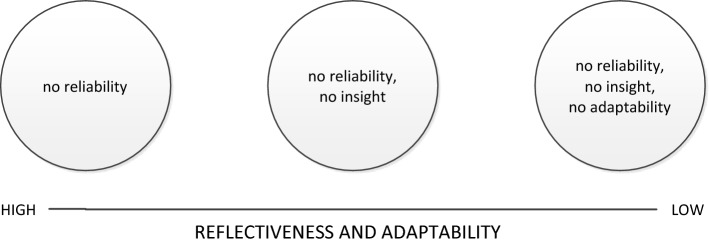
Pre-existing model of profiles of unprofessional behaviour in medical students

Research evidence shows that standardized narratives or *profiles* can effectively represent faculty opinions of residents with borderline performance (Regehr et al. 2012). In our earlier work, we generated such profiles for undergraduate students, in an attempt to aid undergraduate medical teachers to identify unprofessional behaviour (Mak-van der Vossen et al. 2016). This previous study consisted of three methodological steps: firstly, the literature was reviewed to construct a template of unprofessional behaviours. In the second step, students’ unprofessional behaviours, as described by frontline (physician) educators on end-of-attachment evaluation forms, were scored using this template, and subsequently grouped using Latent Class Analysis. In the last step, each class was provided with a description based on the narrative information on the evaluation forms of prototypes of that class. We found three different classes or *profiles* that hypothetically describe the behaviours of students who are cited for unprofessional behaviour. The profiles were: *Poor reliability*, *Poor reliability and poor insight* and *Poor reliability, poor insight and poor adaptability.* Based on the content of the three profiles, the distinguishing variable between the three profiles was described as the *Capacity for self*-*reflection and adaptability* (see Fig. 1 and online appendix #1) (Mak-van der Vossen et al. 2016).

In the current study, we aimed to refine the pre-existing concept (that was created upon opinions of frontline teachers in one institution) by adding perspectives of expert teachers from several medical schools. Thus, we intended to develop a model of unprofessional behaviour profiles in medical students. Adding expert teachers’ perspectives will make it more likely that the final model will be used in practice because of the addition of an experience-based layer to a theoretical concept (Pajares 1992; Turner et al. 2009). An approach to incorporating experts’ perspectives is the use of consensus group methods. Consensus group methods offer a systematic means to gather general agreement, and can also be used to strengthen incomplete empirical evidence from research by adding experience of knowledgeable participants (Humphrey-Murto et al. 2017). The goal of the current study was to refine our earlier research findings by adding systematically collected and synthesized opinions of dedicated experts who represent valuable expertise and multiple viewpoints from different contexts on the evaluation and guidance of students showing unprofessional behaviour. Thus, we aimed to develop a model of profiles of unprofessional behaviour that could help to identify those students who are expected to benefit, and also those who are expected not to benefit from remediation activities.

## Methods

### Study design

We employed Nominal Group Technique (NGT), also called expert panel method, (Humphrey-Murto et al. 2017; Waggoner et al. 2016) and combined this with thematic analysis of expert panel discussions (Ho et al. 2011). In NGT, participants in a meeting share and discuss their perspectives on a certain concept and subsequently independently rank their ideas about this concept. NGT helps to reveal authentic expert opinion without any outside influence, since participants are knowledgeable representatives of the area of inquiry, have practical experience, and come from diverse settings. We selected NGT over other consensus methods (such as a Delphi technique) because it leads to generation of a larger number of ideas (Humphrey-Murto et al. 2017). Furthermore, as participants discuss these ideas among each other, each participant can establish their personal opinions about all introduced ideas based on interaction and discussion with colleagues with similar expertise. A strong facilitator, who should also be a recognized expert in the field, chairs the meeting, mitigating the potential for some participants to unduly dominate the group discussion. The ranking procedure in NGT ensures a democratic result, since final ranking takes place individually and privately.

Using expert panels allowed us to reach our specific aim of refining the pre-existing concept by combining the NGT procedure (leading to generating and ranking of ideas) and thematic analysis of the expert panel meetings (leading to development of a deeper understanding of the ideas). This procedure enhanced our understanding of concepts and terms used, and made it possible to interpret potential differences in contexts between the schools of the participants (Braun and Clarke 2006). Thus, we intended to triangulate quantitative and qualitative data from the expert panel meetings to describe a meaningful whole (Patton 1999).

### Reflexivity

This study was set up using a constructivist paradigm, in which knowledge is seen as actively constructed based on the lived experiences of participants and researchers alike, and co-created by them as the product of their interactions and relationships (Corbin and Strauss 2008). The implication of this choice was that our method had to allow for interaction and discourse between participants, researchers and the studied phenomenon, which led us to choose the NGT and thematic analysis methods, and combine these two (Varpio et al. 2017). Another implication of using the constructivist paradigm is that we must acknowledge that participants and researchers co-created the outcomes of this study: the final results originate from the interaction and discussion among participants and researchers about their shared knowledge and day-to-day experiences. To inform the readers about the knowledge and experience that the authors themselves brought into the study, we share the following with our readers: all authors are education researchers and/or medical educators experienced in teaching and guidance of professional behaviour of medical students. MM, WvM, GC and RAK are medical doctors, AT is an education researcher and AdlC is a linguist. The research question for this study was based on findings from our earlier research, as well as originated from our own teaching experience. To consider our own contribution to the interactive study process we kept an audit trail, which we regularly discussed with each other (Gilgun 2005).

### Procedures and participants

Between October 2016 and January 2018, we collected quantitative and qualitative data through meetings with panels of experts from different medical schools in the Netherlands. In each school one expert panel meeting was organised with the help of a member of the national Special Interest Group on Professionalism of the Netherlands Association for Medical Education (NVMO). These members invited professional behaviour experts at their school, defined as medical educators who had been responsible for the assessment and/or remediation of students with unprofessional behaviour for at least three years. The member asked them if they could mention any other names of experts who would be eligible to participate, so called *snowball sampling* (Berg 1998). These individuals were additionally invited to participate. The NVMO member organized a meeting based on the availability of the experts. The participants were purposively sampled for their knowledge and practical experience, either or both in preclinical and clinical undergraduate medical education, to include a wide range of viewpoints and expertise perspectives from different settings. These experts had been in contact with students who behaved unprofessionally much more frequently than regular *frontline* teachers; the experts are confronted with a selection of students who have shown to behave unprofessionally. Thus, they had developed a specific experience in the guidance of such students. All participants agreed with the procedures, and final scheduling was based on availability. The sample size was not determined ahead of the study. We aimed for sufficiency of the data, meaning that the data should be rich enough to accomplish the aim of the study (Varpio et al. 2017). The sufficiency of the data was determined by reaching consensus in the full research team.

The expert panel meetings were facilitated by a team consisting of two of the researchers (MM, AdlC, WvM, GC and/or RAK), who performed the data collection process in four phases (Humphrey-Murto et al. 2017; Waggoner et al. 2016).

- *Phase 1*: Each meeting started with a presentation of the three profiles of unprofessional behaviour as derived from our earlier research (see Fig. 1, and online appendix 1). Participants were not informed about the results of earlier expert panel meetings at other schools.
- *Phase 2*: Participants were asked to independently and privately generate ideas in response to the following question: “What could we do to improve the profiles to enhance their usefulness for your work?” Each participant wrote down their individual ideas on several post-its.
- *Phase 3*: In a Round Robin format, each individual idea for improvement was shared with the whole group by being read out. The ideas were discussed and clarified within the group, one at a time. All ideas were covered and similar ideas were clustered together into ‘group ideas’ on a flip-over chart. The facilitators ensured that all viewpoints were equally considered, all ideas were discussed and there was agreement about the clustering into group ideas.
- *Phase 4*: The group ideas were given numbers and were written on a new flip-over sheet. Forms with five boxes were handed out so that each participant could write down the five ideas they deemed most important. The boxes were indicated by a five-point Likert type scale, where 5 points = most important and 1 point = least important. Each participant individually and independently (to ensure anonymity) ranked the group ideas into a personal top 5.

Before starting each meeting, participants were informed about the research protocol and ensured of confidentiality, after which their written consent was obtained. All meetings were audio-recorded and transcribed verbatim.

### Data analysis

A. Ranking results

The group ideas and the ranking originating from each expert panel meeting represented the group consensus about refining the pre-existing profiles (see Fig. 1 and online appendix #1). MM and AdlC synthesized the group ideas from all five groups into final ideas, which were confirmed by the full research team. The ranking of the final ideas was established by adding up the rankings from all participants for each group idea, and presented as the percentage of all points.

B. Qualitative data

Two researchers (AdlC and MM) performed thematic analysis of the qualitative data generated from the expert panel meetings (Braun and Clarke 2006), aiming to develop a model that encompassed the attributes nominated by the participants. Using ATLAS-ti, we initially independently coded two transcripts of the group debates in expert panel meetings in an open manner. After several cycles of reading, coding, and discussion, we established a final set of codes and themes. MM coded all transcripts using this set of codes, discussing any difficulties with AdlC. We used memos, diagrams and minutes of research meetings to collect ideas that occurred to us as we moved through the analytic process. By iteratively checking our findings, we ensured that conclusions were grounded in the data. The results were finalized through discussions in the full research team.

C. Developing the pre-existing profiles concept into a final model

Finally, AdlC and MM implemented the ten generated ideas into the pre-existing concept, closely paying attention to the results from the qualitative analysis of the debates. The complete research team discussed the final model, and reached full agreement on the results.

D. Member checking

As a last step the analyses were presented to all participants for a final validation of the adaptations that were made to the pre-existing concept (Varpio et al. 2017). All participants were (by e-mail) asked to give their comments on the results of the study, including the ranking results, thematic analysis and the amended model.

### Results

Data sufficiency was reached after performing five expert panel meetings. These meetings took place at five different medical schools in the Netherlands; a total of 31 faculty participated, including 21 females and 10 males. The backgrounds of the participants were as follows: 9 medical specialists, 6 psychologists, 5 educationalists, 4 general practitioners, 2 registered nurses, 1 psychotherapist, 1 ethics specialist, 1 general physician, and 1 basic medical scientist. The participants had gathered their experience by teaching and assessing students’ professionalism as a frontline teacher for at least 5 years, and furthermore by having oversight over students’ professional development, or by being in remediation or a member of a (professionalism) progress committee for at least 3 years. Each group consisted of five to seven participants. The meetings lasted between 100 and 125 min.

### Primary results

Three types of primary results will be presented: (A) the NGT process ranking results, (B) the thematic analysis of the transcripts, (C) the development of the final model and (D) the validation of results by member checking.

A. NGT process ranking results

**Fig. 2:**
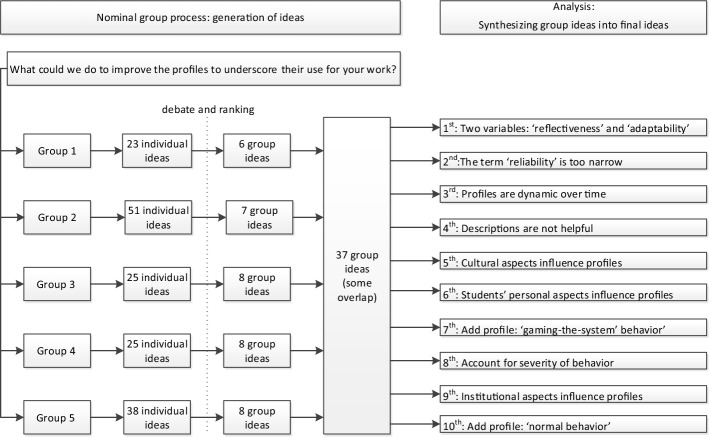
Generated ideas, ranking process and final ideas

The five groups generated 162 individual ideas. After debating and ranking among the participants, only 37 of these ideas got at least one vote. Some of the 37 ideas were very similar, leading to a synthesis of the group ideas from different groups into ten final ideas. Combined, the three most prioritized final ideas received 60% of all points. See Fig. 2 for the idea generating process and ranking into final ideas.

The complete overview of generated individual and group ideas, ranking results, and final ideas can be found in online appendix #2.

B. Thematic analysis of the expert panel meetings

We found three main themes: (1) The profiles and the variable that distinguishes between profiles, (2) The dynamic nature of the profiles over time, and (3) Causal factors for the unprofessional behaviour. These three themes will be discussed below.

1. The profiles and the variable that distinguishes between profiles

In all expert panel meetings participants were generally content with the profiles. They recognized ‘real students’ in them. Participants described the pre-existing profile *no reliability* as ‘normal’ behaviour. Any student, and also any physician, can make an accidental mistake. That is normal, and not problematic if the student listens to feedback and wishes to learn from the mistake. Participants stressed that the professionalism problems that accidentally happen are not limited to reliability concerns, but can be presented by all kinds of unprofessional behaviours, also including disrespectful behaviour, lack of integrity and poor self-awareness.

According to the participants, pre-existing profile *no reliability, no insight* can be divided into behaviour that indicates reflectiveness, but lack of improvement, and behaviour that indicates improvement, without reflectiveness. This way, participants identified an extra behavioural profile in which students seem to display improvement in professional behaviour, without having insight in the way their behaviour relates to the fundamental values of professionalism as adopted by their institution. This behaviour is described as socially desirable: being professional at the right time, the right place, towards the right people. Participants state that it takes time to ultimately recognize this behaviour as unprofessional. They describe the behaviour as *faking* or *gaming*-*the*-*system*. They expressed that this behaviour is worrisome since it is not sustainable behaviour in more challenging circumstances.

Experts recognized the pre-existing profile *no reliability, no insight, no adaptability*: behaviour that indicates no reflectiveness and no improvement of the student over time. Sometimes, behaviours in this profile are so severe that they might threaten patient safety, which thus warrants a punitive approach, instead of a pedagogical, remediating approach.

The distinguishing variable between the profiles, the *capacity to reflect and adaptability*, is not seen as one combined variable but as two distinct variables. Adaptation can be seen with and without reflectiveness, and vice versa. Some students do not have the possibility to adapt, although their reflectiveness is apparent, e.g. when physical or mental health issues or family difficulties play a contributing role. Participants defined the term *adaptability* as the student’s willingness and ability to develop and improve over time. *Reflectiveness* was defined by participants not only as the ability to reflect on own behavior, but also as the willingness to do this.

(2) The dynamic nature of the profiles

Participants stressed that students are not fixed in specific profiles, but the profiles form a time continuum, and student behaviour varies in different times and in changing contexts. This implies that students can move from one profile to another. It also has consequences for the process of diagnosing a profile: Frontline teachers need time to observe the student and to interact with the student to discover the right profile by observing how a student responds to feedback. Based on their perception at the end of their attachment they can ascertain the profile. Remediating faculty need assessments performed by different teachers in different contexts to get the full picture over a period of time. Although they indicated that they often can ‘diagnose’ a profile at once, they always use remediating activities, and the students response to these remediation activities was part of their diagnostic process in confirming the profile.

(3) Causes for unprofessional behaviour

Unprofessional behaviour was attributed to personal circumstances, factors in the educational context and cultural differences.

#### Personal circumstances

Participants indicated that students’ personal constraints influence their professional behaviour. This includes the lack of competencies, such as communication skills or time management and organization skills. Furthermore, internal conditions, such as somatic or psychiatric illness of the student, or external circumstances, e.g. important life events or commitments outside the medical school can contribute to unprofessional behaviour.

#### Circumstances from the educational context

According to the experts, institutional aspects play a role in causing unprofessional behaviour. They mentioned that expectations for professional behaviour are not always made clear to both educators and students. Furthermore, the quality of the educators and the quality of the professionalism assessment method influence students’ professional behaviour. Also, an important factor is that students often experience the educational context as stressful.

#### Cultural differences

Personal and professional values that form the basis for the assessment of professional behaviour differ according to culture, which makes the pre-existing concept difficult to apply to students with non-Western backgrounds. Differences of opinion about unprofessional behaviour, based on different cultural values, can lead to friction about actual behaviours in the workplace. Participants see such differences as difficult to overcome, since a student will not easily change internalized values originating from his or her upbringing. Especially the descriptions of behaviours do not seem to be applicable to non-Western students according to the experts.

In Table 1 the ten final ideas from the NGT-process are illustrated with quotes from the expert panel meetings.

**Table 1:** Three themes, ten final ideas and illustrating quotes from participants

Theme	Rank	Final idea	Quote
The profiles and the variable that distinguishes between the profiles	1	‘Reflectiveness’ and ‘adaptability’ are two distinct distinguishing variables	“Well, maybe there is a class of students who display poor reliability, *good* insight, and poor adaptability. That would mean that we could create four classes of student behaviour, instead of three”
	2	The term ‘reliability’ is too narrow to describe professionalism concerns	“If students fail, and they are referred to us, that can be because they are very arrogant, that can be because they do not engage, that can be because of many other things than not being reliable.”
	4	Leave descriptions of behaviours out	“… you might as well leave the descriptions of behaviours out; the crucial question is: How does the student handle feedback? “
	7	Add profile ‘gaming-the-system behaviour‘	“We see students who have been addressed about their behaviour, and subsequently do exactly what we asked them to do. They pass with desirable behaviours, without being changed fundamentally”
	8	Account for severity of behaviour	“Sometimes you see behaviour that does not fit in class 3; one would say: “I take this student from the clerkship right away, because it is unsafe, this simply cannot be”, and I find profile 3 too mild for that”
	10	Add profile ‘normal’	“I would say…. uhm… profile 1 is the ordinary… uhm… working student, and maybe also the ordinary physician, who now and then put foot in mouth, but if the behaviour is addressed…uhm… that they would know…”
The dynamic nature of the profiles	3	The profiles are dynamic over time	“The fact that someone does not change their behaviour can mean that there are so many things to handle, that, at that point in time, it is just not possible to adapt”
Causes for unprofessional behaviour	8	Cultural aspects influence the profiles	“Many students from non-Dutch origin that I work with will never ask for extra support, because they have not been raised like that. They will listen, and maybe even admit their mistake, but they will never ask for help to improve”
	6	Personal aspects influence the profiles	“What might add is, that for each individual case you look at internal and external factors. Sometimes you see personality disorders. People can have psychiatric illness, or psychological problems. Some people are confronted with all kinds of external hindrances. These are the students who are referred to us. They have been struggling, and at the end of the day they just cannot manage”
	9	Institutional aspects influence the profiles	“Probably, not every teacher is as …uhm….competent as we would want them to be. Do they have the courage that is needed to slow down a student early in the process by paying attention to feedback, and taking time to discover what is happening at that moment?”

C. Development of the final model

We incorporated the ten ideas to improve the profiles and the variables that distinguish between the profiles in the pre-existing concept, paying close attention to the results of the thematic analysis of the transcripts. These amendments are described in Table 2.

**Table 2:** Adaptations that were made to the pre-existing concept as guided by participants’ ideas

Ranking order	Idea	Changes made in the pre-existing concept to create a final model
1	‘Reflectiveness’ and ‘adaptability’ are two distinct distinguishing variables	This prompted to a two-dimensional model including four profiles, distinguished by the variables ‘reflectiveness’ and ‘adaptability’
2	The term ‘poor reliability’ is too limited to describe professionalism concerns	Accordingly, we removed the term ‘poor reliability’
3	The profiles are dynamic over time	We added arrows to illustrate this
4	Leave descriptions of behaviours out	We left the descriptions out
5	Cultural aspects influence the profiles	We acknowledge this in the description of the model, but did not make any changes in the depiction of it, as this influence is applicable to all four profiles of unprofessional behaviour
6	Personal circumstances influence the profiles	This was acknowledged by incorporating the profile ‘struggling behaviour’
7	Add profile ‘gaming the system behaviour’	We added this profile
8	Account for severity of behaviour	We acknowledge that severe unprofessional behaviours can be part of each profile. This did not prompt us to change the model because for such severe unprofessional behaviours both the reflectiveness and adaptability of the student seem to be important
9	Institutional aspects influence the profiles	We acknowledge this in the description of the final model, but did not make any changes in the depiction of it, as this influence is applicable to all four profiles of unprofessional behaviour
10	Add profile ‘normal’	We changed the name of initial profile ‘no reliability’ into ‘accidental unprofessional behaviour’

**Fig. 3:**
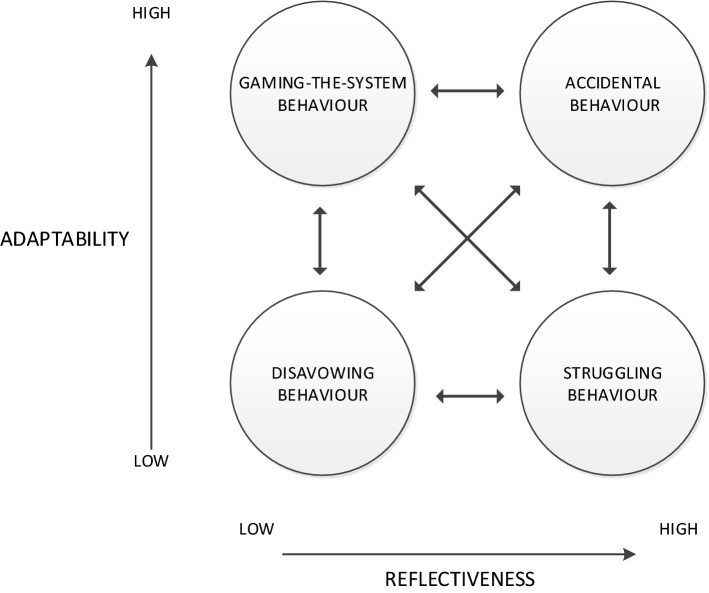
Final model of unprofessional behaviour profiles in medical students

The highest ranked idea from the expert panel meetings was that *reflectiveness* and *adaptability* are two distinct distinguishing variables. This prompted us to devise a two-dimensional model of four profiles distinguished by two variables (see Fig. 3). The pre-existing profile *no reliability* is seen by our participants as normal behaviour, reflecting that unprofessional behaviour can accidentally happen. It is important that the student acknowledges the unprofessional behaviour, and demonstrates that he or she can learn from it. This profile is thus described as *accidental behaviour* in the final model. The pre-existing profile *no reliability, no insight* has been divided in two separate profiles. On the one hand, students’ behaviour that indicates a student’s insight without the possibility to adapt, in the final model described as *struggling behaviour*. On the other hand, students’ behaviour that show improvement, despite lacking insight, in the final model described as *gaming-the-system behaviour*. The pre-existing profile *no reliability, no insight and no adaptability*, describing a student displaying unprofessional behaviour without showing reflectiveness or adaptability over time, has not been changed. In the final model this profile is described as *disavowing behaviour*.

In the expert panel meetings attention has been given to causes for unprofessional behaviour. These ideas were among the lower ranked ideas to improve the pre-existing concept. The revised model does not depict these causes, as they can be equally relevant for any of the profiles.

D. Validation of the results by member checking

After establishing all results, a draft of the results section of the manuscript was sent to the 31 participants of the study. They were asked to individually review the draft, and specially pay attention to Fig. 3. Three participants had left their institution and could not be reached anymore. One participant was not able to review the manuscript due to time constraints. Twenty-five participants returned the email. All but one of them validated the revised model as depicted in Fig. 3. Twenty of them delivered additional remarks on the draft of the result section. These remarks featured (1) the way of indicating that the profiles are dynamic, (2) the interdependency of *reflectiveness* and *adaptability*, and (3) the text of the result section. All remarks were discussed in the full research team. Based on this discussion we decided not to alter Fig. 3. However, we incorporated the experts’ remarks in the results and discussion sections of the manuscript.

## Discussion

The purpose of this study was to refine a pre-existing research-based concept by adding opinions of professionalism experts from different contexts, thus developing a model for unprofessional behaviour profiles of undergraduate medical students. Expert educators participating in expert panel meetings collectively proposed ten ideas to improve the pre-existing concept. The results indicate that the variables discriminating the profiles are *reflectiveness* and *adaptability*. Furthermore, two additional profiles emerged: *gaming*-*the*-*system behaviour* and *struggling behaviour.*

Experts stressed the fluidity of the profiles, which means that students can move from one profile to another over time. Surprisingly, specific narrative descriptions of unprofessional behaviours appeared not to be important to the experts. We used these findings to construct a final model of four profiles and two distinguishing variables. This final model should guide medical educators to recognize unprofessional behaviours of undergraduate students, thus facilitating the identification of students who underperform in the competency of professionalism. The final model could also support the decision-making process to remediate or dismiss learners from further training.

The two variables discriminating between the profiles appeared to be *reflectiveness* and *adaptability*. This confirms earlier findings that reflective ability plays a role as a determinant for describing the thresholds between pass and fail for professional behaviour (Kalet et al. 2016, 2017; Hoffman et al. 2016) We add to these earlier findings that *adaptability* is also an important guiding factor in the decision-making process on remediation strategies or dismissal. A question that still remains is: “Are these two variables independent, or do they influence each other?”

Two new profiles were described. The first new profile was the profile of *gaming*-*the*-*system* behaviour. Experts expressed that this behaviour is difficult to detect, since teachers obviously find it difficult to recognize this behaviour. *Gaming*-*the*-*system behaviour* seems to be the display of desired professional behaviour based on external norms, without having personally internalized the values of professionalism. This brings up the question if *faking* or *gaming*-*the*-*system* behaviour is unprofessional, or a threshold phase in the learning process (Neve et al. 2016). Or is it a way that students protect themselves from burn-out in the highly challenging environment of medical education? Our findings indicate the importance of the students’ awareness of this situation. *Fake it till you make it* can be an effective strategy, as long as the learner is aware that it is a means to an end (Larson and Yao 2005; Patel et al. 2018). The second new profile, *struggling behaviour* is widely acknowledged in the medical education literature about burnout (Dyrbye et al. 2010). Also in this case, the student’s awareness of the situation seems crucial for further development.

Experts stressed that students can move from one profile to another over time. Our findings indicate that reflectiveness and adaptability are important aspects to consider in making decisions about seriousness of the professionalism deficiency. Students’ response to feedback, and improvement thereafter is part of establishing the fitting profile. Teachers typically take a snapshot, act accordingly, and later re-evaluate the student’s performance to ascertain or modify the profile chosen. Possibly, not only students’ profiles are dynamic, but also educators’ opinions about them. This warrants the programmatic assessment method, in which performance is assessed over a period of time, by combining assessments of different educators (Van der Vleuten and Schuwirth 2005). This also implies that remediation activities should be part of the normal educational process, and integrated in the medical education program (Kalet et al. 2017).

Descriptions of specific behaviours turned out to not be discriminative. Possibly the narrative descriptions that came with the initial profiles were too detailed and context-specific. In contrast with frontline teachers, who seem to focus on behaviours, expert teachers pay more attention to students’ reflectiveness and improvement. This finding is a contribution to the existing literature about detecting underperformance, in which behaviours, attributions for behaviours and consequences of behaviours have been described (Teherani et al. 2009; Ginsburg et al. 2009; Guerrasio et al. 2014). Our findings confirm that reflectiveness is related to professionalism concerns (Hoffman et al. 2016). Accidental unprofessional behaviour is not seen as problematic, but a lack of reflectiveness and a lack of improvement after feedback on observed unprofessional behaviour are seen as indicators that a student needs remediation.

The pre-existing concept was based on frontline (physician)-teachers’ evaluations of professional behaviour on evaluation forms, and the final model of profiles is based on opinions of expert faculty. We hypothesize that the differences between the pre-existing concept and the final model could be explained by the different approaches of frontline (physician)-educators and experts to students’ unprofessional behaviour, in several phases of the process of recognizing unprofessional behaviour (see Table 3).

**Table 3:** Different approaches to students’ unprofessional behaviour by frontline (physician)-teachers and by experienced professionalism educators

Phase in the diagnostic process	(Physician)-educators (who delivered data for the pre-existing concept)	Expert PB-educators (who delivered data for the final model)
Observing	Observe students for a short time	Observe students for a longer time
Identifying	Primarily identify behaviours as reliability problems	Identify unprofessional behaviour as a lack of reflectiveness and improvement
Acknowledging	Need time to acknowledge unprofessional behaviour	Acknowledge unprofessional behaviour instantly, and confirm afterwards
Explaining	Account for students’ intentions	Account for personal, contextual and cultural causes
Remediating	Strive to improve actual professional behaviour	Strive to stimulate longitudinal professional development

We used the rankings of the expert panel meetings to generate consensus on ideas, and the thematic analysis to understand and describe the underlying reasons and mechanisms for the amendments in order to come to the model of unprofessional behaviour profiles. Using the NGT method in combination with thematic analysis of the expert panel meetings allowed us to refine and develop the pre-existing concept in three ways. (1) We were able to incorporate practical experience from faculty in the pre-existing concept, which originated from empirical evidence. (2) This experience was derived from professionalism experts, while the pre-existing concept was based on information from frontline medical teachers. (3) Furthermore, experience from different medical schools supported the research findings from one institute (VUmc). These three aspects make it likely that the findings, reduced to a model, display the reality of educational practice, and will be applied by medical educators (Pajares 1992; Turner et al. 2009).

### Limitations

A limitation of the method we used is that the five expert panel groups did not interact with each other, and thus participants were not able to comment on ideas from other groups. Nevertheless, the 1st and 3rd ranked ideas came forward from all groups, and the 2nd ranked idea from four of the five groups, indicating the relevance of these ideas. We addressed this limitation by performing a member checking of the combined results of all expert panel meetings. Another limitation is that the results were influenced by the different educational cultures prevalent in the participants’ institutions. An example is that the influence of cultural differences on professional behaviour was especially indicated by the expert groups from those medical schools that are known for having students from diverse (international) backgrounds. To account for any blind spots, we incorporated all ten group ideas into the final model.

Furthermore, as we limited this study to medical schools in The Netherlands, results are not plainly generalizable to an international context.

### Implications for education and future research

The profiles can be useful for frontline teachers because identification of a certain profile can help to decide if a student needs to be referred for further guidance after the teacher’s course has been finished. Frontline teachers should not only focus on reliability issues, but also on a student’s reflectiveness and adaptability, which are seen as essential aspects of professionalism by expert faculty. The profiles can be useful for individuals with remediation oversight to follow the student’s development after remediation has been applied, especially students’ reflectiveness and adaptability.

Educational researchers have to investigate if the profiles are a means to determine effective remediation. *Reflectiveness* and *adaptability* could possibly be incorporated as thresholds for remediation in frameworks that are under development (Ellaway et al. 2017; Kalet et al. 2017). Based on the findings of our study, we postulate the following remediation strategies for each of the profiles that need to be studied further for outcome effectiveness. For the profile of *accidental behaviour* the student needs to become aware that anyone can make a mistake based on the combination of personal, contextual and cultural causal factors, and that the goal is to let the individual learn from mistakes, support each other in doing so, and collectively learn from these accidental unprofessional behaviours. For students who display *gaming*-*the*-*system behaviour* the relevance of professional behaviour needs to be made clear, so that they can internalize the professionalism values. The student with *struggling behaviour* needs support for the internal or external causal factor for the unprofessional behaviour. This might include guidance from resources outside the medical school. The *disavowing behaviour* seems to be the most challenging to remediate. These students initially need to develop reflective skills, and be motivated to try out alternative behaviour based on the feedback provided to them. We intend to address these hypotheses in a future study. Furthermore, it would be interesting if new descriptions or vignettes that fit the profiles could be developed through research. The hypothesized differences between frontline teachers and expert teachers as described in Table 3 also need to be confirmed by research.

## Conclusion

This study used expert educators’ opinions on the evaluation of professional behaviour in undergraduate medical education to refine a pre-existing concept of profiles of unprofessional behaviour in medical students and to develop it into a final model. While evaluating professional behaviour, expert faculty want to follow students over time to discover students’ adaptability and reflectiveness. Reflectiveness and improvement over time are considered more important than displayed unprofessional behaviours. This implies that remediation of unprofessional behaviour should primarily focus on these two aspects, and is preferably designed as a part of the regular medical curriculum. The empirical findings of the current study can have consequences for the choice of remediation strategies and could add to frameworks on success and failure being developed in medical education systems, aiming to define expertise to conduct effective remediation.

## Electronic supplementary material

Below is the link to the electronic supplementary material.

Supplementary material 1 (DOCX 18 kb)

Supplementary material 2 (PDF 51 kb)
