# Supplementary material for: Developing a two-dimensional model of unprofessional behaviour profiles in medical students
Source: Adv Health Sci Educ Theory Pract. 2018 Nov 1;24(2):215–32. doi: 10.1007/s10459-018-9861-y (PMC6484089; doi:10.1007/s10459-018-9861-y)
Supplement: Supplementary file 1 — Supplementary material 1 (DOCX 18 kb) [file 10459_2018_9861_MOESM1_ESM.docx]

**Additional file #1: Initial three profile concept**

Profile descriptions from a study of patterns in the unprofessional behavior of undergraduate medical students, VUmc School of Medical Sciences, Amsterdam, the Netherlands, 2012-2014

| **Profile** | poor reliability | poor reliability  +  poor insight | poor reliability  +  poor insight  +  poor adaptability |
| --- | --- | --- | --- |
| **Distinguishing factor** | **Capacity of self-reflection and adaptability** | | |
| **Profile description** | A student from class 1 does not obey rules and regulations of the school. The student does not inform teachers and peers about his/her activities. When receiving feedback the student admits that his behavior was unprofessional. The student often asks for help to improve. | A student from class 2 does not actively participate in study groups or clerkships, and is often late or absent. Communication with peers and teachers is inadequate. The student relies on peers, sometimes resulting in plagiarism. When this behavior is addressed this student does not recognize the feedback, but is willing to accept a different viewpoint. In coaching conversations a student from class 2 exhibits good intentions and willingness to change. | A student from class 3 seems to have problems in interpersonal communication and teamwork. This student often does not understand information given by others, which leads to misunderstandings. Peers and teachers ̶ sometimes patients ̶ feel that they are not always treated respectfully by this student, but the student does not recognize their feelings. A student from class 3 does not accept the teachers’ feedback and does not improve. The student is not able to formulate learning goals and often does not accept an offered coaching trajectory. |
