# Supplementary material for: Developing a two-dimensional model of unprofessional behaviour profiles in medical students
Source: Adv Health Sci Educ Theory Pract. 2018 Nov 1;24(2):215–32. doi: 10.1007/s10459-018-9861-y (PMC6484089; doi:10.1007/s10459-018-9861-y)
Supplement: Supplementary file 2 — Supplementary material 2 (PDF 51 kb) [file 10459_2018_9861_MOESM2_ESM.pdf]

| 10 final ideas | # points | # points | #points | # points | # points | total | % of all points |
|---|---|---|---|---|---|---|---|
| Capacity to reflect and adaptability are two independent variables | 21 | 17 | 6 | 42 | 27 | 113 | 24,9 |
| 'Reliability' should be replaced by 'unprofessional behavior' | 0 | 24 | 24 | 19 | 31 | 98 | 21,6 |
| Profiles are dynamic over time | 7 | 16 | 12 | 3 | 15 | 53 | 11,7 |
| Narrative descriptions are not helpful, can be removed | 29 | 16 | 0 | 0 | 6 | 51 | 11,3 |
| Personal aspects influence profile | 0 | 0 | 19 | 0 | 22 | 41 | 9,1 |
| Cultural aspects influence profile | 17 | 0 | 2 | 12 | 4 | 35 | 7,7 |
| Account for severity of behavior | 0 | 20 | 0 | 6 | 0 | 26 | 5,7 |
| Add profile: gaming | 10 | 0 | 6 | 7 | 0 | 23 | 5,1 |
| Institutional aspects influence profile | 0 | 12 | 0 | 0 | 0 | 12 | 2,6 |
| Add profile : normal | 0 | 0 | 0 | 1 | 0 | 1 | 0,2 |
| | | | | | | 453 | 100,0 |

| university | #participants | # ranking points | # individual ideas | # ideas after ranking | descriptions of 37 ideas after ranking, partly overlapping | final ideas | points/all groups | points/participants/group |
|---|---|---|---|---|---|---|---|---|
| | | | | | Extra profile: reflection, no adaptation | two variables: reflectiveness and adaptability | 21 | 3.5 |
| | | | | | Add to profile 2: student sees the incident, but not the pattern | narrative descriptions | 14 | 2.3 |
| | | | | | Add non-verbal behaviors to all profiles | narrative descriptions | 15 | 2.5 |
| | | | | | Cultural aspects | cultural aspects | 17 | 2.8 |
| | | | | | Extra profile: 'gaming' professionalism by showing desired behaviors | add profile: gaming | 10 | 1.7 |
| 1 | 6 | 90 | 23 | 6 | Profiles are dynamic | profiles are dynamic | 7 | 1.2 |
| | | | | | Add profile: patient safety threatened | add profile: unsafe | 20 | 2.9 |
| | | | | | Latent variable: adaptability is part of each profile | two variables: reflectiveness and adaptability | 17 | 2.4 |
| | | | | | Instutional definition of professionalism should be clear | institutional aspects | 9 | 1.3 |
| | | | | | Profiles are dynamic | profiles are dynamic | 16 | 2.3 |
| | | | | | Reliability should be defined | reliability too narrow | 24 | 3.4 |
| | | | | | Teachers' skills/behavior/viewpoints influence profile | institutional aspects | 3 | 0.4 |
| 2 | 7 | 105 | 51 | 7 | Leave descriptions out, it's about handling feedback | narrative descriptions | 16 | 2.3 |
| | | | | | Poor reliability' is too narrow | reliability too narrow | 13 | 2.6 |
| | | | | | Profiles form a continuum | profiles are dynamic | 12 | 2.4 |
| | | | | | Reliabily, is applicable to preclinical, not to clinical students | reliability too narrow | 11 | 2.2 |
| | | | | | Adaptability should be distinguished in will to adapt, and skill to adapt | two variables: reflectiveness and adaptability | 6 | 1.2 |
| | | | | | Personal limitations of student | personal aspects | 11 | 2.2 |
| | | | | | Extra profile: gaming the system | add profile: gaming | 6 | 1.2 |
| | | | | | Personal apects can be temporarily | personal aspects | 8 | 1.6 |
| 3 | 5 | 75 | 25 | 8 | Cultural aspects are important | cultural aspects | 2 | 0.4 |
| | | | | | Add severity of behavior | add profile: unsafe | 6 | 1.0 |
| | | | | | Reflexivity and adaptability are two independent variables | two variables: reflectiveness and adaptability | 21 | 3.5 |
| | | | | | Cultural aspects are important | cultural aspects | 12 | 2.0 |
| | | | | | Add profile: will/no skill | two variables: reflectiveness and adaptability | 21 | 3.5 |
| | | | | | Add profile: gaming behavior | add profile: gaming | 7 | 1.2 |
| | | | | | Add profile: normal | add profile : normal | 1 | 0.2 |
| | | | | | Term 'reliability' is too narrow | reliability too narrow | 19 | 3.2 |
| 4 | 6 | 90 | 25 | 8 | Profiles develop over time | profiles are dynamic | 3 | 0.5 |
| | | | | | Term 'reliability' is too narrow | reliability too narrow | 31 | 4.4 |
| | | | | | Profiles are not cumulative | profiles are dynamic | 15 | 2.1 |
| | | | | | Extra profile: will, no skill | two variables: reflectiveness and adaptability | 8 | 1.1 |
| | | | | | Leave descriptions out | narrative descriptions | 6 | 0.9 |
| | | | | | Students' development is important | personal aspects | 18 | 2.6 |
| | | | | | Students' initiative is important | personal aspects | 4 | 0.6 |
| | | | | | Cultural aspects are important | cultural aspects | 4 | 0.6 |
| 5 | 7 | 105 | 38 | 8 | Reflectiveness and coachability are two independent variables | ability | 19 | 2.7 |
| 5 groups | 31 participants | 465 ranking points | 162 ideas | 37 ideas after ranking | 37 ideas after ranking | 10 different ideas | total 453 ranking poi | |
